# Emerging proteomics biomarkers and prostate cancer burden in Africa

**DOI:** 10.18632/oncotarget.16568

**Published:** 2017-03-25

**Authors:** Henry A. Adeola, Jonathan M. Blackburn, Timothy R. Rebbeck, Luiz F. Zerbini

**Affiliations:** ^1^ International Centre for Genetic Engineering and Biotechnology, Cape Town, South Africa; ^2^ Department of Integrative Biomedical Sciences, Faculty of Health Sciences, University of Cape Town, Cape Town, South Africa; ^3^ Institute of Infectious Disease & Molecular Medicine, Faculty of Health Sciences, University of Cape Town, Cape Town, South Africa; ^4^ Harvard T.H. Chan School of Public Health and Dana-Farber Cancer Institute, Boston, Massachusetts, USA

**Keywords:** proteomics, biomarker, prostate cancer, Africa, mass spectrometer

## Abstract

Various biomarkers have emerged *via* high throughput omics-based approaches for use in diagnosis, treatment, and monitoring of prostate cancer. Many of these have yet to be demonstrated as having value in routine clinical practice. Moreover, there is a dearth of information on validation of these emerging prostate biomarkers within African cohorts, despite the huge burden and aggressiveness of prostate cancer in men of African descent. This review focusses of the global landmark achievements in prostate cancer proteomics biomarker discovery and the potential for clinical implementation of these biomarkers in Africa. Biomarker validation processes at the preclinical, translational and clinical research level are discussed here, as are the challenges and prospects for the evaluation and use of novel proteomic prostate cancer biomarkers.

## INTRODUCTION

Prostate cancer (PCa) is one of the leading causes of cancer death in men globally [1]. Factors responsible for aggressive or indolent phenotypes of PCa are poorly understood. The chances of developing PCa significantly increases after the age of 40 years [2]. However, even without any form of therapy, PCa often runs a protracted natural history and many men die with it rather than from the disease [3]. Over a million new cases are reported annually according to the GLOBOCAN/ IARC 2012 databases; PCa is the fifth leading cause of cancer death in men globally, accounting for up to 307,000 deaths annually [4]. Among cancers in Africa, PCa was reported to have the highest incidence (59,493), mortality (42,802) and 5 year prevalence rate (155,028).

**Figure 1:**
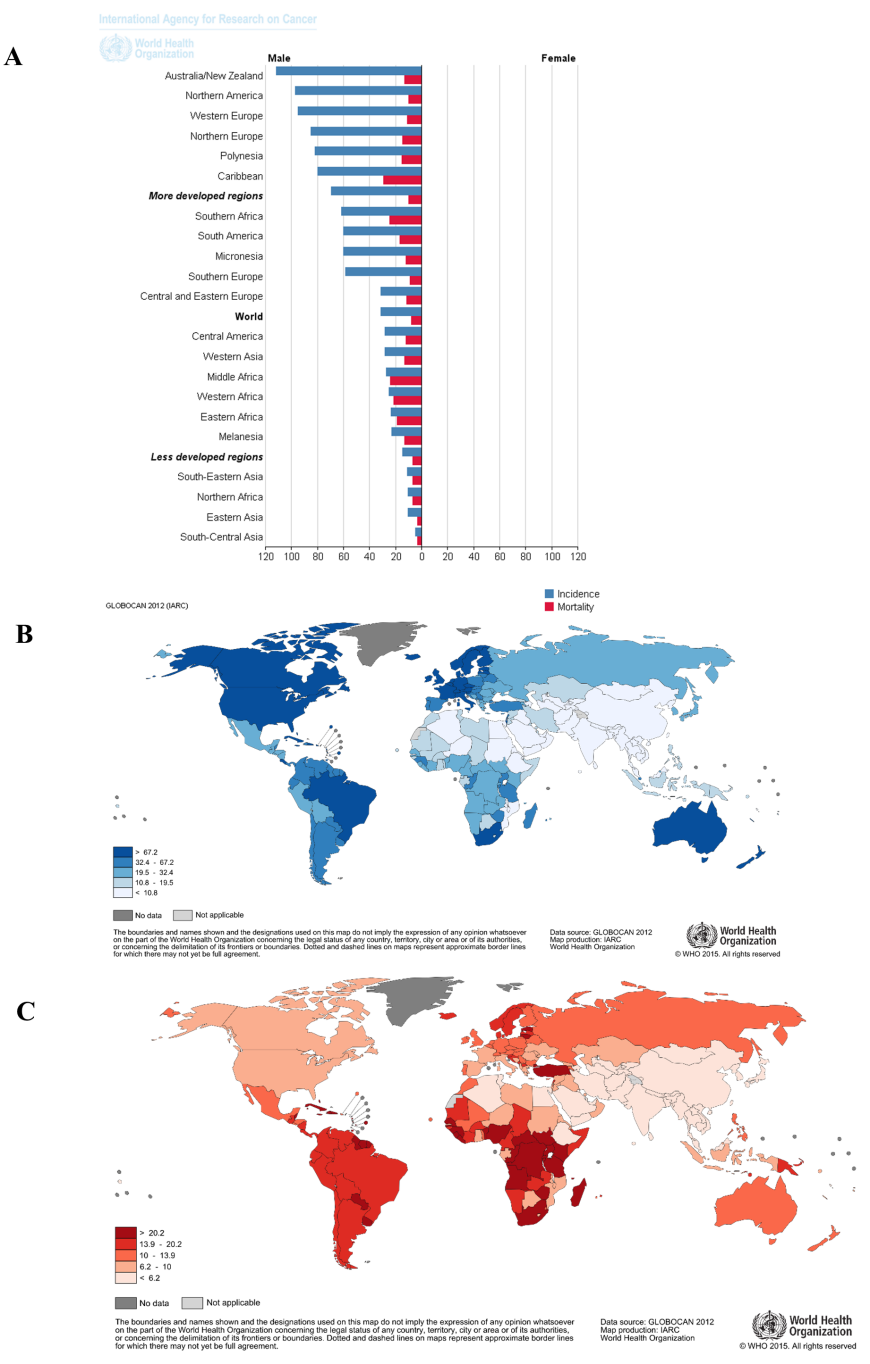
Global epidemiology of prostate cancer showing high burden of prostate cancer in Africa **A**. A bar graph showing highest incidence and mortality of PCa in Eastern, Middle, Western and Southern regions of Africa as well as the Caribbean regions. **B**. and **C**. are maps demonstrating high incidence and mortality of prostate cancer in sub-Saharan Africa respectively. Even with a high incidence in South Africa, there is still a relatively high mortality of prostate cancer in this region in comparison to the western world. (Maps and bar graphs were adapted from the online cancer fact sheets of the WHO/IARC GLOBOCAN database 2012 at http://globocan.iarc.fr/Pages/fact_sheets_cancer.aspx).

This high incidence, mortality and 5-year prevalence trend is similar in sub-Saharan Africa, Southern Africa, or the Republic of South Africa. This clearly contrasts with the situation in the developed world, where high incidence and low mortality reflect the impact of early diagnosis and prompt treatment. In most situations, cases are diagnosed at advanced stages in Africa and the mortality and incidence rates are almost at par with the situation in the Western, Middle, and Eastern Africa (Figure 1A) [5]. Owing to a relatively higher level of development and infrastructure in the Republic of South Africa, PCa incidence is quite high compared to the rest of Africa. However, mortality rates in this region are high as well. The fact that South Africa has a high incidence rates similar to those in North America, Western Europe and Australia but a high mortality rate comparable to most other sub-Saharan African countries (Figure 1B & 1C) suggests that despite a relatively better diagnostic infrastructure compared with other parts of Africa, limited manpower and resources has complicated the management of the huge burden of diagnosed PCa cases [5].

The current conventional regimen for PCa diagnosis, which includes PSA measurement, is unreliable in the diagnostic grey area of the reference ranges (2-10ng/mL). Hence, identifying biomarkers which tally accurately with disease risk and staging, as well as providing for evidence based treatment, is key in the reduction of PCa burden in men of African descent. Although PCa may be more aggressive in men of African descent [6], lack of access to care and delay in diagnosis has obfuscated evidence of a biologically more aggressive disease in African descent men. Among speculated causes of aggressive PCa disease in African men, only age [7, 8] and genetic factors [9, 10] are incontrovertible. Men over the age of 40 and men of African descent are at greater risk of developing PCa compared to their Caucasian counterpart. Men of Asian origin have the least risk of developing PCa, albeit their risk has been reported to increase when they migrate to North America [11]. Few aetiologic and risk factors have been suggested to be associated with PCa development. Modifiable risk factors for PCa have been difficult to identify. Obesity, smoking, alcohol consumption, androgens, diet, diabetes mellitus, and hereditary factors, *inter alia*, have not been consistently associated with PCa etiology [11]. PCa susceptibility gene loci on 1q24-25 (HPC1), 8p22 (MSR1), 1q25 (RNASEL), and 17p11 (ELAC2) have been recognized by genome wide association studies (GWAS) [12, 13]. Some biological pathways that have been enriched for genetic variants in PCa GWAS were JAK2, IGF-1, prolactin, and androgen signaling pathways [14]. A better understanding of the role of these putative genetic markers is needed among African men with PCa.

## CURRENT DIAGNOSTIC BIOMARKERS OF PCA

Despite the benefit that the discovery of PSA provided in PCa detection, it remains an imperfect biomarker and there is room to add omics-based biomarkers to improve PCa detection. A number of cancer-related biomarkers of PCa have been identified that may play a role in early PCa detection or prognosis, including: PTEN, PI3K, PCA7 gene panel, PSGR, MME, PSCA, PCA3, TMPRSS2-ERG gene fusion, CD98, EPCA, CD276, prostate-specific membrane antigen (PSMA), caveolin-1, EN-1, and annexin A3 [15].

### Serum markers

Acid Phosphatase (ACPP) is one of the oldest biomarkers used for PCa diagnosis in serum, however the drawback of this biomarker is that ACPP is expressed by both normal and malignant prostatic tissues, as well as extraprostatic tissues [16]. Prostate-Specific Antigen (PSA), also known as kallekrein 3 (KLK3) is the most widely used serum biomarker of PCa and has tremendously improved the diagnosis of PCa. PSA screening was widely adopted in the USA in early 1990s as gold standard investigation for PCa [17]. However, recent evidence indicates that PSA falls short in its diagnostic ability in the lower reference ranges (2-10ng/mL). This led to its contraindication in the USA for men greater than 75 years in 2008 [18]; and for all men in 2012 [19]. Even though highly sensitive, it is not so specific and has led to a high false positive rate, false negatives and overtreatment of PCa patients. Despite this down side to PSA, very few biomarkers are currently poised to replace PSA in clinical practice [20, 21]. To improve the diagnostic ability of PSA, several related parameters such as PSA doubling time, PSA velocity, free-to-total PSA ratio and prostate health index (PHI) have been explored [22].

### Tumour markers

Prostate cancer antigen 3 (PCA3) which is otherwise known as DD3 or differential display clone 3 is a noncoding mRNA which is found to be highly abundant in malignant prostatic tissues in comparison to benign [23, 24]. It is the most widely used non-PSA based biomarker for PCa diagnosis [25]. One limitation of PCA3 test is that it is dependent on the urinary PSA transcript expression level. TMPRSS2-ERG gene fusions (Transmembrane Protease, Serine 2- ETS fusion) are members of the ETS family of genes, and can be highly expressed in malignant prostatic tissue but not expressed in benign tissue. Emerging research evidence suggests TMPRSS2-ERG fusions are unlikely to be good predictors of PCa outcomes or aggressiveness. They are expressed in only about 50% of all PCa patients, and varies substantially by race, with African-Americans having much lower staining rates [26]. It suffers the same drawback as PCA3 in that it depends on urinary PSA transcript levels for meaningful interpretation of result. Alpha methylacyl-CoA racemase (AMACR) is a highly sensitive and specific diagnostic biomarker often used in PCa tissues. Low levels of AMACR in biopsy tissues have been associated with biochemical recurrence and PCa metastasis [27].

**Figure 2:**
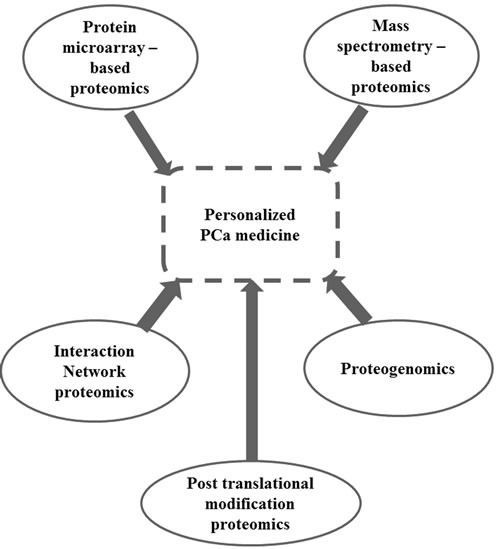
Role of proteomics in personalized medicine of prostate cancer Various proteomics approaches have improved the individualization of prostate cancer therapy. An integrative approach using these proteomics methodologies would improve the identification of proteomics biomarkers of prostate cancer. As shown here, proteomics approaches such as MS-based proteomics, protein microarrays-based proteomics, interaction network proteomics, proteogenomics and well as posttranslational modification proteomics have all been of great benefit in biomarkers development for personalized/individualized therapy of Prostate cancer.

Emerging proteomics approaches such as mass spectrometry (MS), protein microarrays, interactomics, proteogenomics as well as posttranslational modification proteomics have also been very useful in the development of biomarkers for personalized/individualized therapy of PCa (Figure 2). It is plausible that interplay of various other omics-based approaches would benefit personalized PCa therapies. For example, a recently described classification of the seven possible subtypes of PCa [28, 29] based on TMPRSS2: ERG translocations may represent a more useful molecular classification of PCa (in terms of therapeutic options) than histologic classification. Even though these emerging biomarkers (PCA3, TMPRSS2-ERG, and AMACR) have been validated in Western populations [30, 31], fewer such studies have primarily focused on African-American or African populations [32, 33]. A few other good reviews of genomic biomarkers of PCa can be found elsewhere [34–36].

## PROSPECTS FOR PROTEOMICS IN PCA BIOMARKER RESEARCH

The field of proteomics is a high-throughput approach to large-scale identification of the full complement of proteins in an organism, tissue, cells or body fluid. These methods are also potentially able to investigate the functional states of proteins including, post-translational modifications, protein-protein interactions; and protein interaction with other biomolecules such as carbohydrates, lipids and other metabolites. Proteomics can provide insight into 3-D protein structures, alternative splicing events, as well as aiding genome annotation. There are variations to the proteome of a cell depending on the time point, stage of disease, diet and a host of other factors. Currently, proteomics has been employed to identify cancer-related signatures between disease and healthy cohorts of patient [37–42]. Notably, the most common proteomics methodologies are MS-based proteomics and protein microarray technology based proteomics. Using these methodologies, a gamut of proteomics biomarkers of PCa have already been identified [43–49], and some were demonstrated to potentially predict progression and aggressiveness of PCa [50–52]. However, successful application of omics based approaches is heavily dependent on available bioinformatics and computational biology resources, which remain limited in Africa.

## BIOSAMPLE SOURCES FOR PROTEOMICS BIOMARKERS OF PCA

The preclinical phase of biomarker developed is the foundation upon which translational and clinical validation can be built. Even though tissue-based proteomics has been widely performed [53–56], body fluid-based proteomics, albeit challenging, offers a non- or minimally-invasive alternative [57, 58] with comparable or even superior diagnostic accuracy to tissue-based proteomics, combined with greater suitability for large-scale screening or early detection methods. Potentially suitable body fluids for PCa biomarker discovery include urine, blood and semen. Liquid biopsy has been used for diagnosis of various cancer types [59–63], including PCa [64, 65]. Additionally, this technique has been found useful in cancer patient stratification, monitoring and screening [66].

As an ultrafiltrate of blood, urine possesses analogous protein profiles as are found in peripheral blood and provides a usable catalog of proteins for interpretation of pathophysiologic events in the human body [67]. Sampling urine as compared to blood or prostatic tissue biopsy is a less invasive approach for PCa diagnosis and treatment monitoring. The service of skilled personnel is not required for urine sample collection. In addition to this, urine is abundantly available and permits repeated sampling [68]. Notably, the urinary proteome is less complex compared with the blood proteome and is comparatively stable, the proteins having undergone any likely proteolysis in blood or during storage in the bladder [68]. Hence, there is no need to use protease inhibitors during urine storage [68]. Despite the fact that urine is a very promising disease biomarker source, a major caveat is that its composition is variable depending on the time of the day, dietary intake and the state of health of the individual. Many urinary biomarkers of various diseases have been previously described [69], albeit only a few of these biomarkers have entered into clinical use [70–73].

Considering its extensive contact with body structures, human blood is an attractive source for biomarker discovery. Increased discohesiveness of cancerous tissues sometimes makes tumour cells more mobile and is transported in the blood stream as tumour markers. Even though limited in volume, it is the most frequently used sample for clinical diagnosis of many disease conditions. Blood samples have been used to identify biomarkers of many human diseases including: Alzheimer's disease [74], Parkinson's disease [75], breast cancer [76], preeclampsia [77], and PCa [23]. Importantly, it is also known that disease conditions such as cancer are able to stimulate humoral immune response leading to the generation of auto-antibodies [78]; and this has been demonstrated in many different human cancers, including renal [79], colorectal [80], lung [81], and PCa [82–86]. These autoantibodies generated, have potential theranostic utilities for cancer diagnosis and therapeutic vaccine target development.

## PROTEOMICS BIOMARKER DISCOVERY IN AFRICA

Both urine and blood are very attractive preclinical biomarker sources for PCa in Africa. PCa proteomics has been carried out in African-American populations [87, 88] but there are a limited number of studies among indigenous men of African descent. One such study identified 82 novel potential urinary biomarkers of PCa in a heterogeneous cohort of 45 South African patients composed of Indigenous, Mixed-Ancestry and Caucasian African PCa patients *via* label-free MS [89]. Among these 82 identified biomarkers, nine biomarkers were identified that suggested racial differences among the ethnicities in the cohort. Verification and prevalidation of the 82 biomarkers using both experimental ‘parallel reaction monitoring’ and *in silico* computational methods enabled identification of the top performing 12 potential biomarkers [90], ready for translation through a large scale, multiplatform and multicenter targeted proteomics validation study. Furthermore, relating to the ability of cancers to stimulate humoral immune response in the body, 41 novel potential serological autoantibody profiles have been identified in a South African PCa patient cohort (*N* = 67) [91]. These identified serological autoantibody responses could potentially be used in the construction of mini-arrays as point of care diagnostic tools as well as for therapeutic vaccine development for PCa in Africa. Many of these potential biomarkers have been reported in literature as being associated with other diseases, PCa as well as cancers in other human body organs; however, many are still yet to be described in PCa [89]. A comprehensive list which highlights the emerging potential biomarkers of PCa from those studies in terms of their source, methods used for their discovery and how far down the biomarker discovery pipeline they have moved, is provided in Tables 1A, 1B, 1C, 1D. Even though several potential preclinical biomarkers of PCa have been discovered in Western studies, very few studies have identified and validated novel potential biomarkers of PCa in Africa.

**Table 1A:** List of urinary and serological proteomic biomarkers discovered in prostate cancer in a South African cohort

Potential PCa Proteomic biomarkers	Method used	Biospecimen used	Prevalidated	Ethnic trend
Alpha-2-macroglobulin	MS	Urine	N	N
Alpha-actinin-1	MS	Urine	Y	N
Alpha-N-acetylglucosaminidase	MS	Urine	N	N
Apolipoprotein A-II;Truncated apolipoprotein A-II	MS	Urine	N	N
Apolipoprotein B-100;Apolipoprotein B-48	MS	Urine	N	N
Apolipoprotein C-III	MS	Urine	N	N
Basement membrane-specific heparan sulfate proteoglycan core protein	MS	Urine	N	N
Beta-defensin 1	MS	Urine	N	N
C4b-binding protein alpha chain	MS	Urine	N	N
Cadherin-11	MS	Urine	N	N
Carbonic anhydrase 1	MS	Urine	N	N
Carbonic anhydrase 2	MS	Urine	N	N
Carboxypeptidase N catalytic chain	MS	Urine	Y	N
Cathepsin Z	MS	Urine	Y	N
CD59 glycoprotein	MS	Urine	N	N
Collagen alpha-1(VI) chain	MS	Urine	N	N
Collagen alpha-1(XII) chain	MS	Urine	N	N
Collagen alpha-2(I) chain	MS	Urine	N	N
Collagen alpha-3(VI) chain	MS	Urine	N	N
Complement component C8 alpha chain	MS	Urine	N	N
Complement factor H	MS	Urine	N	N
Cystatin-M	MS	Urine	N	N
Dihydrolipoyllysine-residue succinyltransferase component of 2-oxoglutarate dehydrogenase complex, mitochondrial	MS	Urine	N	N
Epididymal secretory protein E1	MS	Urine	N	N
Fibrillin-1	MS	Urine	N	N
Flavin reductase (NADPH)	MS	Urine	N	N
Galectin-1	MS	Urine	N	Y
Ganglioside GM2 activator	MS	Urine	N	N
Gastrotropin	MS	Urine	N	Y
Glutaredoxin-1	MS	Urine	N	N

**Table 1B:** List of urinary and serological proteomic biomarkers discovered in prostate cancer in a South African cohort

Potential PCa Proteomic biomarkers	Method used	Biospecimen used	Prevalidated	Ethnic trend
Glyceraldehyde-3-phosphate dehydrogenase	MS	Urine	N	N
Haptoglobin	MS	Urine	Y	N
Haptoglobin-related protein	MS	Urine	N	N
Heat shock protein HSP 90-beta	MS	Urine	N	Y
Hemoglobin subunit alpha	MS	Urine	N	N
Hemoglobin subunit beta	MS	Urine	N	N
Histone H1.5	MS	Urine	N	N
Ig delta chain C region	MS	Urine	N	N
Ig Heavy chain V-III region ZAP	MS	Urine	N	Y
Ig kappa chain V-I region BAN	MS	Urine	N	N
Inter-alpha-trypsin inhibitor heavy chain H1	MS	Urine	N	N
Inter-alpha-trypsin inhibitor heavy chain H2	MS	Urine	N	N
Inter-alpha-trypsin inhibitor heavy chain H3	MS	Urine	N	N
Lactotransferrin	MS	Urine	N	N
Leukocyte-associated immunoglobulin-like receptor 1	MS	Urine	N	N
Lithostathine-1-alpha	MS	Urine	N	N
Ly-6/neurotoxin-like protein 1	MS	Urine	N	N
Lysozyme C	MS	Urine	N	N
Mannan-binding lectin serine protease 2 A chain	MS	Urine	N	N
Mannosyl-oligosaccharide 1,2-alpha-mannosidase IA	MS	Urine	N	Y
Monocyte differentiation antigen CD14	MS	Urine	N	N
Myocilin	MS	Urine	Y	Y
N-acetylmuramoyl-L-alanine amidase	MS	Urine	Y	N
Neutrophil gelatinase-associated lipocalin	MS	Urine	N	N
Nidogen-1	MS	Urine	Y	N
Non-secretory ribonuclease	MS	Urine	N	N
Osteopontin	MS	Urine	N	N
Pancreatic alpha-amylase	MS	Urine	N	N
Plasma kallikrein	MS	Urine	N	N
Plastin-2	MS	Urine	N	N
Platelet glycoprotein Ib Alpha chain; Glycocalicin	MS	Urine	N	Y
Polyubiquitin-C	MS	Urine	N	N
Pregnancy zone protein	MS	Urine	Y	N
Pro-epidermal growth factor;Epidermal growth factor	MS	Urine	N	N
Prostaglandin-H2 D-isomerase	MS	Urine	N	N

**Table 1C:** List of urinary and serological proteomic biomarkers discovered in prostate cancer in a South African cohort

Potential PCa Proteomic biomarkers	Method used	Biospecimen used	Prevalidated	Ethnic trend
Prostate-specific antigen	MS	Urine	Y	N
Prostatic acid phosphatase;PAPf39	MS	Urine	Y	N
proteasome inhibitor P131 subunit	MS	Urine	N	Y
Protein S100-A9	MS	Urine	N	N
Ribonuclease pancreatic	MS	Urine	N	N
Roundabout homolog 4	MS	Urine	N	N
Saposin-D	MS	Urine	N	N
Serum paraoxonase/arylesterase 1	MS	Urine	N	N
SH3 domain-binding glutamic acid-rich-like protein 3	MS	Urine	N	N
SLAIN motif-containing protein 1	MS	Urine	Y	Y
Tenascin	MS	Urine	N	N
Trefoil factor 1	MS	Urine	N	N
Trefoil factor 2	MS	Urine	N	N
Trefoil factor 3	MS	Urine	N	N
Uteroglobin	MS	Urine	N	N
Vitamin K-dependent protein S	MS	Urine	Y	N
WAP four-disulfide core domain protein 2	MS	Urine	N	N
BORIS BO	CAA	Blood	N	N
CAMEL	CAA	Blood	N	N
CAML1	CAA	Blood	N	Y
CCDC33	CAA	Blood	N	N
CDK2	CAA	Blood	N	Y
CEACAM1 Isoform 1	CAA	Blood	N	N
COL6A1	CAA	Blood	N	Y
CSAG2	CAA	Blood	N	N
CT47.11	CAA	Blood	N	N
DDX53	CAA	Blood	N	N
DPPA4	CAA	Blood	N	N
EGFR	CAA	Blood	N	N
FES	CAA	Blood	N	N
FGFR2	CAA	Blood	N	N
GAGE1	CAA	Blood	N	N
GAGE5	CAA	Blood	N	N
LDHC	CAA	Blood	N	N

**Table 1D:** List of urinary and serological proteomic biomarkers discovered in prostate cancer in a South African cohort

Potential PCa Proteomic biomarkers	Method used	Biospecimen used	Prevalidated	Ethnic trend
MAGEA11	CAA	Blood	N	N
MAGEB1	CAA	Blood	N	N
MAGEB5	CAA	Blood	N	N
MAGEB6	CAA	Blood	N	N
MAPK3	CAA	Blood	N	Y
NY-ESO-1	CAA	Blood	N	N
OIP5	CAA	Blood	N	Y
p53	CAA	Blood	N	N
p53 C141Y	CAA	Blood	N	N
p53 K328R	CAA	Blood	N	N
p53 L344P	CAA	Blood	N	N
p53 Q136X	CAA	Blood	N	N
p53 S15A	CAA	Blood	N	Y
p53 S392A	CAA	Blood	N	N
p53 S46A	CAA	Blood	N	N
p53 T18A	CAA	Blood	N	Y
PBK	CAA	Blood	N	Y
PRKCZ	CAA	Blood	N	N
RAF	CAA	Blood	N	N
ROPN1A	CAA	Blood	N	Y
SPANXA1	CAA	Blood	N	N
SSX2A	CAA	Blood	N	N
TKTL1 (Isoform a)	CAA	Blood	N	N
ZNF165	CAA	Blood	N	N
CAA= Cancer antigen array; MS= Mass spectrometry; Y=Yes; N=No				

## CURRENT APPROACHES TO PCA PROTEOMICS BIOMARKER DEVELOPMENT

**Figure 3:**
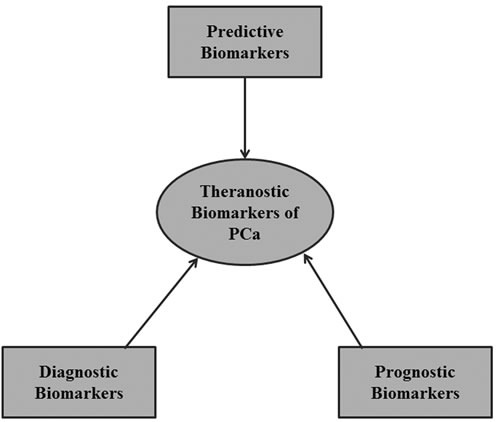
A theranostic approach to biomarker development A single theranostic biomarker is capable of functioning as a diagnostic, prognostic and predictive biomarker simultaneously.

The essence of translational research in the biomarker pipeline is to create a bridge from bench-derived or preclinical biomarkers to clinical utility. The clinical validation pipeline is comprised of various stringent phases through which preclinical biomarkers are tested before they can be certified for clinical utility. These biomarkers are applicable to various stages of PCa diagnosis and treatment as described below. Many of the currently emerging biomarkers have theranostic capabilities, implying that they possess predictive, diagnostic and prognostic potential [92–94] (Figure 3). Such double-edged “theranostic” biomarkers, which are capable of aiding diagnosis and well as serving as a means of treatment are very much needed in Africa where early diagnosis, treatment costs and patient compliance play a major role in PCa management outcome. Considering that the burgeoning biomarkers of PCa in the literature are poorly validated [95], more effort is needed to establish innovative ways to improve the success of emerging biomarkers through the validation phases and into clinical application.

The need for systematic validation of proteomics biomarkers and lack of standardization of validation methods among researchers [71, 96, 97] has led to the establishment of the Early Detection Research Network (EDRN) and the Prospective Specimen Collection, Retrospective Blinded Evaluation (PRoBE) collaboration with the National Cancer Institute (NCI) [25, 98]. One important identified cause of unsuccessful biomarker validation is the lack of concordant experimental outcome from independent research groups [25]. Prensner *et al* therefore suggested a three-phased biomarker validation pipeline, starting with a discovery phase, followed by validation in a retrospective cohort, and then final validation in a prospective cohort [24]. Mandrekar *et al* emphasized that critical planning is essential in biomarker validation design and that the use of prospective randomized controlled trials as a gold standard enables a distinction between prognostic and predictive biomarkers [99]. Analytic validation, clinical qualification/validity and clinical utilization has been recommended as vital steps in the rigorous evaluation of biomarkers and surrogate endpoints in chronic diseases [72, 99].

Using these biomarker validation steps, features including high-grade prostatic intraepithelial neoplasia (HGPIN), PSA level, apoptosis and proliferation may be considered as surrogate endpoint biomarkers of PCa [100]. In addition, Brown *et al* suggested that bone-related peptides like N- and C-terminal peptide fragments of type I collagen, and bone-specific alkaline phosphatase can be used as endpoint biomarkers in PCa [101]. Despite the fact that the drug development pipeline is well established, biomarker validation pipeline is still a controversial subject among biomarker researchers. Through the efforts of the EDRN, Pepe *et al* have developed a 5-phased biomarker validation pathway [97, 102] that extends the ideas of Prensner *et al* [24]: The first stage is the preclinical exploratory phase where promising potential biomarkers are identified; Phase II involves clinical assay and validation to identify disease establishment; Phase III is a retrospective longitudinal study for preclinical detection of disease; Phase IV involves a prospective screening of the characteristics and extent of disease; and Phase V is aims at cancer control by assessing the effect of screening with biomarker on the burden of disease in the population (Table 2). Several standard guidelines for reporting and evaluating biomarker studies have emerged: reporting recommendation for tumour markers (REMARK); biospecimen reporting for improved study quality (BRISQ); minimum information about a microarray experiment (MIAME); standard for reporting diagnostic accuracy (STARD); and the level of evidence (LoE) based tumor marker guideline (TMUG) proposed by the American Society of Clinical Oncology (ASCO) in 1996 [72]. These guidelines have promoted transparency and rigour in the way biomarker discovery is being reported and evaluated; albeit most are yet to be implemented in African cancer biomarker studies.

**Table 2:** The Early Detection Research Network (EDRN) 5-phase biomarker validation pipeline for the identification and validation of potential biomarkers for cancer control (Pepe *et al* 2001 [97])

Phase	Activity	Expected outcome
**I**	Preclinical exploratory phase	Promising potential biomarkers are identified
**II**	Clinical assay and validation	Identification of disease establishment
**III**	Retrospective longitudinal study	Preclinical detection of disease
**IV**	Prospective screening	Characteristics and extent of disease
**V**	Assessment of effect of screening with biomarker on the burden of disease	Cancer control

## CHALLENGES AND PROSPECTS OF PCA PROTEOMICS BIOMARKER DISCOVERY IN AFRICA

It is clear that the burden PCa on the African continent has increased significantly over the recent years, with the highest mortality rates reported in sub-Saharan Africa [6]. It has also been demonstrated that PCa incidence rates in African-American and West African populations differ, even though they share a common genetic ancestry [7, 9]. The bases for such disease disparities are largely unknown, and it has been difficult to develop state-of-the-art research on prostate tumorigenesis and biology in Sub-Saharan Africa. Barriers to knowledge about PCa in Sub-Saharan Africa include sociocultural issues such as poor funding [102, 103], insufficient manpower [102] and skilled health personnel [104], poor access to healthcare [10], religious and cultural beliefs [105], lack of well-updated cancer registries [106], poor research and healthcare infrastructure [102, 107], low educational level [108], prevalence of infection [109, 110], poor governance structures and fiscal policies [111]. Although these factors are not unique to the African continent, the underlying genetic, hereditary and environmental basis of PCa aggressiveness in men of African descent still warrants further research.

The advent of proteomics and other high throughput omics-based technologies has highlighted the need for computational biology, as well as for state-of-the-art banking of experimental biospecimens. Improper documentation and storage of biologic specimen may result in skewed biochemical inferences, histopathologic analysis and predicted therapy. Hence, a good specimen biorepository is an essential infrastructure for development of high throughput omics based personalized medicine in Africa. Most ground breaking projects in the field of molecular biology such as the Human Genome Project (HGP) [112], The Cancer Genome Atlas (TCGA) [113], Human Proteome Project (HPP) [114] and Chromosome Centric Human Proteome Project (CHPP) [115] have benefitted immensely from specimen biorepositories. However, in Africa, it is apparent that there is a limited biobanking capacity and that procedures such as fresh snap frozen tissue sampling cannot be performed in most places because liquid nitrogen is largely unavailable. Furthermore, there has traditionally been a paucity of bioinformatics infrastructure and bioinformaticians in Africa, although this is now being addressed through National Institutes of Health (NIH)-funded training and capacity development initiatives, such as the H3ABionet consortium. The National Cancer Institute has described a biorepository as human specimen collection including relevant data for the purpose of research, and subject to relevant processes, ethics and policies [116, 117]. Despite the gamut of biorepositories established in the Americas, Europe, Asia and Australia [118–120], very few such biobanks have been established in sub-Saharan Africa [121]. Most biorepositories in Africa are established within investigator's research group and most are yet to be standardized and centralized. Several challenges have plagued biorepository development and regulation in sub-Saharan Africa [121–124], albeit modest progress has been made in a few sub-Saharan African countries. Notably, the emergence of the NIH and Wellcome Trust-funded H3Africa consortium, has improved the centralization and standardization of biospecimen collection in Africa [124]. Further collaborative effort is required by clinicians and scientists to standardize and improve the development of a centralized biorepository in Africa, such that high throughput ‘omics technologies can be backed by adequate research materials/resources, enabling them to play a central role in improving our current understanding of PCa in Africa.

*In silico* prevalidation is required in early phase of biomarkers development study [21, 90], to optimize potential biomarkers prior to large scale studies. The cost implication and infrastructure needed for large scale validation of omics based biomarkers can be prohibitive [125], hence collaborative efforts such as the EDRN are necessary to achieve this goal [98]. In addition, standard validation procedures often require validated surrogate prognostic biomarkers that have been tested in multiple phase III trials [126]. Due to prohibitive costs, only important proteins or antibodies from the discovery phase would typically be validated [127]. Proteogenomic integration is thus an important multipronged approach to identify viable diagnostic and treatment signatures for PCa. For example, androgen receptors (AR) and EGFR has been identified as correlating with PCa progression using gene expression data integrated with protein interaction networks [128]. In line with an integrated proteogenomic approach, other emerging liquid biopsy biomarkers such as long non-coding RNA and exosomes holds future prospects for PCa [129]. Sixteen putative proteomics biomarkers of PCa validated on a well annotated tissue microarray (TMA) containing ca. 2500 PCa samples has been used to establish a prediction nomogram for PCa [130]. Several genomic classifiers have also emerged for clinical metastasis and biochemical recurrence, as well as for patient stratification [131]. Hitherto, most discovered biomarkers have focused on diagnostic rather than prognostic and predictive potentials [25]. As emphasized earlier, there is an urgent need for larger biorepository establishment to facilitate disease biomarker discovery and validation [25]. Emerging biomarkers such as circulating tumour cells (CTC) in blood may enable the evaluation of disease prognosis and survival [132]. Advances in PCa biomarker development has resulted in the clinical utility of laboratory tests such as Prolaris score, Oncotype Dx, Confirm MDx, Prostarix and 4K score [133]. These tests have helped to improve diagnosis of PCa and decipher the level of evidence (LoE) assigned for biomarker validation [134]. Urine and blood sample testing for biomarkers is a reasonable approach with high prospect for mass screening of populations for disease [135], particularly in African populations that are at greater risk of developing aggressive PCa.

## CONCLUSIONS AND RE-COMMENDATIONS

This review has demonstrated the prospects for discovery and validation of potential biomarkers in Africa. Currently available biomarkers and biomarkers sources were highlighted, as well as the application of high throughput methodologies for candidate biomarker discovery. Being a continent with a huge burden of cancer and mostly populated by low and middle income countries (LMIC), several challenges have been identified that militate against the routine application of proteomics to PCa research. The most promising urinary biomarkers which require further validation are the top 12 identified by targeted proteomics [90]; while the best serological biomarkers are the top 41 tumour associated antigens [91]. All potential urinary and serological biomarkers that demonstrated ethnic trends are also worth investigating further for potential used for personalized management of PCa in Africa. There is a pressing need to develop cancer research initiatives and collaborations with partners within and outside of Africa. Potential PCa biomarkers discovered in African studies requires research and systematic validation as practiced in the developed world, with urine and blood based theranostic biomarkers offering a multi-pronged approach to diagnosis and treatment of PCa in Africa. Ethnic-tailored biomarkers for PCa management are required if personalized medicine is to become a reality among African populations. Collaborative research directed towards understanding the potential role of PCa immunotherapy in African patients should also be encouraged, both locally and internationally. Importantly, diagnostic focus should be redirected towards less invasive approaches to PCa management, to improve early screening and intervention in Africa. It is also suggest that a centralized specimen and proteome repository should be built for PCa in Africa, as this would provide a resource for better research into PCa burden among men of African descent.

## Abbreviations

ASCO: American Society of Clinical Oncology
BRISQ: Biospecimen Reporting for Improved Study Quality
CHPP: Chromosome Centric Human Proteome Project
CTC: Circulating Tumour Cells
EDRN: Early Detection Research Network
GLOBOCAN: Global Cancer Statistics Database
GWAS: Genome-Wide Association Studies
HGP: Human Genome Project
HGPIN: High-Grade Prostatic Intraepithelial Neoplasia
HPP: Human Proteome Project
IARC: International Agency for Research on Cancer
LoE: Level of Evidence
MIAME: Minimum Information about a Microarray Experiment
NCI: National Cancer Institute
PCa: Prostate Cancer
PHI: Prostate Health Index
PRoBE: Prospective Specimen Collection, Retrospective Blinded Evaluation
REMARK: Reporting Recommendation for Tumour Markers
STARD: Standard for Reporting Diagnostic Accuracy
TCGA: The Cancer Genome Atlas
TMA: Tissue Microarray
TMUG: Tumor marker guideline
